# Impaired myogenic tone in mesenteric arteries from overweight rats

**DOI:** 10.1186/1743-7075-9-18

**Published:** 2012-03-16

**Authors:** Karen L Sweazea, Benjimen R Walker

**Affiliations:** 1 School of Nutrition and Health Promotion, Arizona State University, Phoenix, AZ, USA; 2 Vascular Physiology Group, Department of Cell Biology and Physiology, University of New Mexico Health Sciences Center, Albuquerque, NM, USA

**Keywords:** Myogenic tone, Calcium, Oxidative stress, Nitric oxide, Mesenteric arteries

## Abstract

**Background:**

Rats fed high fat (HFD) or high sucrose (HSD) diets develop increased adiposity as well as impaired vasodilatory responsiveness stemming from oxidative stress. Moreover, HFD rats become hypertensive compared to either control (Chow) or HSD fed rats, suggesting elevated vascular tone. We hypothesized that rats with increased adiposity and oxidative stress demonstrate augmented pressure-induced vasoconstriction (i.e. myogenic tone) that could account for the hypertensive state.

**Methods:**

Male Sprague-Dawley rats were fed Chow, HFD or HSD for 6 weeks. The effects of oxidative stress and endogenous nitric oxide on myogenic responses were examined in small mesenteric arteries by exposing the arteries to incremental intraluminal pressure steps in the presence of antioxidants or an inhibitor of nitric oxide synthase, LNNA (100 μM).

**Results:**

Contrary to the hypothesis, rats fed either HSD or HFD had significantly impaired myogenic responses despite similar vascular morphology and passive diameter responses to increasing pressures. Vascular smooth muscle (VSM) calcium levels were normal in HFD arteries suggesting that diminished calcium sensitivity was responsible for the impaired myogenic response. In contrast, VSM calcium levels were reduced in HSD arteries but were increased with pre-exposure of arteries to the antioxidants tiron (10 mM) and catalase (1200 U/mL), also resulting in enhanced myogenic tone. These findings show that oxidative stress impairs myogenic tone in arteries from HSD rats by decreasing VSM calcium. Similarly, VSM calcium responses were increased in arteries from HFD rats following treatment with tiron and catalase, but this did not result in improved myogenic tone. Nitric oxide is involved in the impaired myogenic response in HFD, but not HSD, rats since inhibition with LNNA resulted in maximal myogenic responses at lower intraluminal pressures and VSM calcium levels, further implicating reduced calcium sensitivity in the impaired response.

**Conclusion:**

The impaired myogenic responses observed in isolated arteries from HSD and HFD rats are attributed to changes in VSM calcium signaling.

## Background

Increased adiposity is associated with the development of diabetes, hypertension and cardiovascular disease [1-3]. Oxidative stress is a complication of increased adiposity and can directly induce hypertension through superoxide (O_2_^•-^)-mediated scavenging of the endogenous vasodilator nitric oxide (NO) [4,5]. Results from prior studies show that feeding rats a high fat (HFD) or high sucrose (HSD) diet increases adiposity and oxidative stress contributing to impaired endothelium-dependent vasodilation [6]. HFD-fed rats additionally develop significant fasting hyperglycemia compared to both Chow and HSD-fed rats (Chow: 74.86 ± 5.6; HSD 75.4 ± 6.2; HFD 102.4 ± 5.0 mg/dl; [6]). As expected, blood pressure is also significantly elevated in HFD fed rats (153.5 ± 2.4 vs. 137.5 ± 2.7 mmHg for Chow-fed rats; *p <*0.05) [7]. Although blood pressure in the HSD fed rats was not measured in prior studies, it is expected to be elevated as a result of the observed impaired vasodilatory responses [6]. To test this, systolic blood pressure of the HSD-fed rats was measured in the present study.

Myogenic tone is the ability of the vascular smooth muscle (VSM) layer of a blood vessel to constrict when the vessel is radially stretched due to intravascular pressure [8] and is dependent on an influx of calcium into the VSM [9]. Although myogenicity is an inherent property of VSM, the endothelium may produce factors that modulate myogenic tone. Since endothelium-dependent vasodilation is impaired in mesenteric arteries from both HSD and HFD rats [6], myogenic responses to increasing intraluminal pressures might be predicted to be elevated in these animals. In the intact mesenteric arterial vasculature, myogenic tone is more pronounced in vessels from the distal segments. This response of small arteries has been shown to be entirely dependent on the VSM since removal of the endothelium has no impact on the response [10]. The present study was designed to examine the effects of oxidative stress on the myogenic response of small mesenteric arteries from overweight animals in contrast to studies in the literature that have mainly focused on the effects of obesity and overt diabetes on these blood vessels. In one study, for example, oxidative stress was shown to augment myogenic tone in gracilis muscle arteries from obese Zucker rats [11]. Our hypothesis, therefore, was that increased oxidative stress leads to enhanced myogenic tone in arteries from overweight rats fed either high sucrose or high fat diets.

## Methods

All protocols and surgical procedures used in this study were reviewed and approved by the Institutional Animal Care and Use Committees of the University of New Mexico School of Medicine (Albuquerque, NM, USA) and Arizona State University (Tempe, AZ, USA). All chemicals were purchased from Sigma Aldrich.

### Experimental groups

Adult male Sprague-Dawley rats (140-160 g body weight, Harlan Industries) were divided into three groups and fed either normal chow (Chow), high sucrose (HSD), or high fat (HFD) diets. The Chow diet contained in kcal: 18.9% protein, 57.33% carbohydrates (% sucrose NA), and 5% fat (2018; Harlan Teklad). The high sucrose diet was comprised of 20% protein, 70% carbohydrates (34.5% sucrose), and 10% fat (D12450B; Research Diets, Inc., New Brunswick, NJ). The high fat diet contained 20% protein, 20% carbohydrates (6.8% sucrose) and 60% fat (D12492; Research Diets, Inc.). Rats were maintained on the respective diets for 6 weeks and the food was replaced every 3-4 days to prevent spoiling. All animals had access to food and water *ad libitum *and were housed in identical cages in the same animal facility and exposed to a 12:12h light dark cycle. Systolic blood pressure of a separate cohort of HSD rats was measured by tail plethysmography for comparison to previously published values for rats fed Chow and HF diets [7].

### Morphology of mesenteric arteries

A segment of the mesenteric arcade and associated small intestine was extracted from deeply anesthetized rats (sodium pentobarbital, 200 mg/kg, i.p.) and placed in 4% formalin prior to embedding in paraffin. Sections (5 μm) were collected onto glass slides and stained using a commercially available kit according to the manufacturer's protocol (Cat. HT25A; Sigma Aldrich, St. Louis, MO). This kit stains elastic fibers black, collagen fibers bright pink and smooth muscle fibers muted pink to brown. Sections were counterstained with eosin. Images were collected using a Nikon Eclipse E400 microscope equipped with a Nikon DS-Fi1 camera controlled by NIS-Elements software (Nikon, Melville, NY).

### Myogenic tone

The mesenteric arcade was removed following a midline laparotomy from deeply anesthetized rats (sodium pentobarbital, 200 mg/kg, i.p.). The isolated arcade was pinned out in a Silastic coated dissection dish filled with ice-cold HEPES buffer (in mM: 134.4 NaCl, 6 KCl, 1 MgCl_2_, 1.8 CaCl_2_, 10 HEPES, 10 glucose, pH 7.4) and fifth-order mesenteric resistance arteries (~1 mm length; 80-120 μm, i.d.) were isolated. Arteries were transferred to a HEPES filled vessel chamber (Living Systems, CH-1, St. Albans, VT), cannulated with glass pipettes, and secured with silk ligature. The vessels were pressurized to a resting pressure of 60 mmHg with a servo-controlled peristaltic pump (Living Systems Instrumentation, St. Albans, VT) and the chamber was placed on a microscope stage for continuous measurement of the inner diameter of the vessels using video microscopy and edge-detection software (IonOptix, Milton, MA). Vessels were superfused with warm aerated physiological salt solution (PSS; 37°C) containing (in mM): 129.8 NaCl, 5.4 KCl, 0.5 NaH_2_PO_4_, 0.83 MgSO_4_, 19 NaHCO_3_, 1.8 CaCl_2_, and 5.5 glucose at a rate of 10 mL/min. Vessels were exposed to the vasoconstrictor phenylephrine (PE; 10^-6^M) followed by the vasodilator acetylcholine (ACh; 10^-6^M) in the superfusate prior to each experiment to verify viability.

Following equilibration of isolated mesenteric arteries in heated PSS for 30-min (37°C; 10 mL/min), VSM were loaded with the cell-permeant ratiometric calcium-sensitive fluorescent dye fura 2-AM (2 μM) in 4 mL of HEPES buffer for 45 minutes in the dark at room temperature. Arteries were washed for 15 minutes with warmed, aerated PSS to allow for hydrolysis of AM groups by intracellular esterases and to remove excess fura 2-AM from the superfusate. Arteries were then superfused for 1 hour with either a control PSS solution (*n = *5-9), PSS with the addition of the general NOS inhibitor Nω-nitro-L-arginine (LNNA; 100 μM, *n *= 6 per group), or combined treatment with the SOD mimetic 4,5-dihydroxy-1,3-benzene-disulfonic acid (tiron; 10 mM) and the H_2_O_2 _scavenger catalase (1200 U/mL; *n = *5-6 per group). Myogenic responses to increasing intraluminal pressures (20 to 120 mmHg, 3 min at each 20 mmHg step) were measured by recording the inner diameter of the blood vessel. Blood vessel inner diameters were continuously monitored from bright field (red light) images using video microscopy and edge-detection software (IonOptix, Milton, MA). Fura loaded vessels were alternatively excited at 340 and 380 nm, and the respective 510-nm emissions were quantified using a photomultiplier tube (IonOptix) and recorded using IonWizard software (version 4.4, IonOptix). Following the myogenic response curves, arteries were superfused for 30-min with calcium-free PSS containing (in mM): 129.8 NaCl, 5.4 KCl, 0.5 NaH_2_PO_4_, 0.83 MgSO_4_, 19 NaHCO_3_, 5.5 glucose, and 3 EGTA and the measurement of responses to increasing intraluminal pressures was repeated to obtain the passive inner diameter at each step from which percent vasoconstriction was calculated.

Pre-exposure of arteries to LNNA or tiron and catalase were used to determine the role of NOS and ROS, respectively, in the vasoconstrictor responses of arteries. Additional experiments to assess the combined effects of LNNA and tiron and catalase on vasoconstrictor responses were also performed. Pretreatment of arteries with LNNA significantly increased the basal tone of arteries from HSD and HFD rats whereas pretreatment with tiron and catalase had no significant effect (data not shown).

### Statistics

Data are expressed as means ± SEM. Passive inner diameter responses to increasing intraluminal pressures were measured in arteries following treatment with calcium-free PSS and were compared across groups by two-way repeated measures ANOVA (RM-ANOVA). Percent vasoconstriction was calculated as the percent difference of inner diameter observed at each pressure step vs. calcium free values. Data for all myogenic response curves were arcsine transformed to approximate a normal distribution and analyzed using two-way RM-ANOVA. Where significant effects occurred, individual groups were compared using Student-Newman-Keuls post hoc analyses. Results from within group analyses were used to determine the pressure at which no further significant increases in myogenic tone were observed (i.e. the plateau or maximal response). Changes in VSM calcium were determined by the difference in F_340_/F_380 _emissions at each pressure step compared to values obtained at 20 mmHg. A probability of ≤0.05 was accepted as statistically significant for all comparisons.

## Results

**Figure 1:**
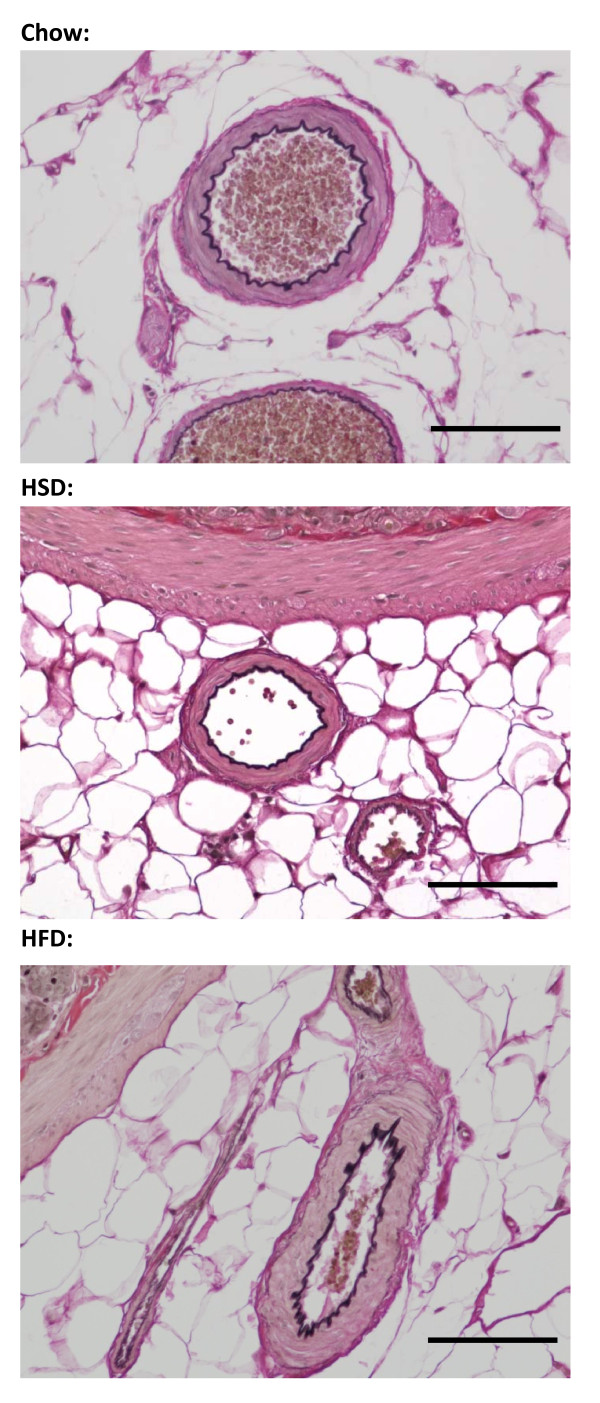
**Representative images showing the morphology of mesenteric arteries from rats in all three dietary groups**. Elastic fibers are stained black, collagen fibers bright pink, and smooth muscle fibers muted pink to brown. Cross sections were viewed at 200x. Scale bar: 50 μm.

**Figure 2:**
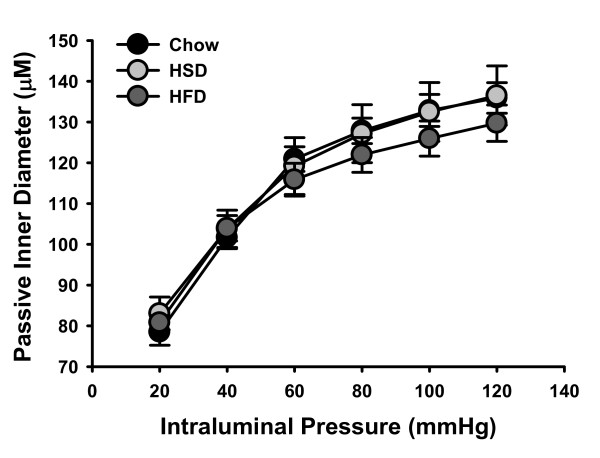
**Passive responses to increasing intraluminal pressures**. Following the myogenic response curves, all vessels were superfused with a calcium-free PSS solution and the curve repeated to obtain the passive diameter response. There were no significant differences in the passive inner diameter responses to increasing intraluminal pressures between experimental groups. Data are expressed as mean ± SEM.

**Figure 3:**
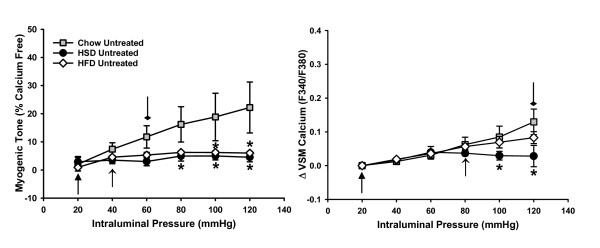
**Comparison of myogenic responses in isolated mesenteric arteries from Chow (*n *= 5), HSD (*n *= 8) and HFD (*n *= 9) fed rats**. Plateaus in responses at which point no further increases were seen were determined from the two-way RM-ANOVA within group analyses and are indicated by arrows: diamond-head, Chow; triangle-head, HSD; simple arrow, HFD. *p<0.05 from Chow untreated arteries. Data expressed as mean ± SEM.

Both HSD and HFD rats were overweight compared to their Chow-fed counterparts with HFD rats gaining the most weight (in grams Chow: 330.9 ± 6.0; HSD: 361.3 ± 5.2*; HFD: 376.5 ± 4.9*^,#^; where *p < 0.05 vs. chow, ^#^*p *< 0.05 vs. HSD). In contrast to previous findings from HFD rats [7], HSD-fed rats do not become hypertensive (143.7 ± 2.6 mmHg). Morphological analyses of mesenteric arteries also showed similar elastic, collagen and smooth muscle staining between groups (Figure 1). In addition, the passive diameter responses to increasing intraluminal pressures were not significantly different between groups (Figure 2). Although arteries were morphologically similar, myogenic responses were significantly diminished in arteries from both HSD and HFD fed rats compared to arteries from Chow rats (Figure 3). The myogenic response reached a plateau at 60 mmHg in arteries from Chow rats at which point the response no longer significantly increased. In contrast, arteries from both HSD and HFD rats reached a plateau much earlier (20 and 40 mmHg, respectively; Figure 3). VSM calcium responses in arteries from Chow rats did not reach a plateau at any intraluminal pressure step whereas arteries from HSD rats showed a plateau matching that of the myogenic response (20 mmHg; Figure 3). In contrast, VSM calcium levels in arteries from HFD rats were not significantly different from the Chow fed rats, suggesting that decreased contractile calcium sensitivity of the VSM may be responsible for the diminished myogenic response in this group. This is further supported by the maximum VSM calcium levels being reached at 80 mmHg whereas the maximum myogenic tone was reached at a much lower pressure of 40 mmHg (Figure 3).

**Figure 4:**
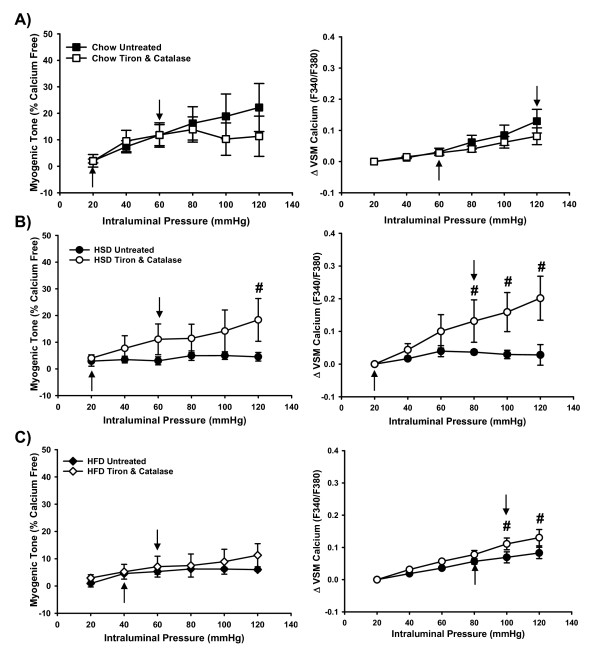
**Role of oxidative stress in the impaired myogenic responses observed in arteries from Chow (*n *= 5), HSD (*n *= 5) and HFD (*n *= 6) rats**. Experimental vessels were exposed to the combined antioxidants tiron (10 mM) and catalase (1200 U/mL). Maximum responses at which point no further increases were seen are indicated by arrows and were determined from the two-way RM-ANOVA within group analyses. Data from untreated HSD and HFD arteries repeated from Figure 2 for comparison. *#p <*0.05 from respective HSD or HFD untreated arteries at the same pressure step. Data expressed as means ± SEM.

Figure 4A shows pre-treatment of arteries from Chow rats with tiron and catalase lowered the maximal myogenic response (to 20 mmHg from 60 mmHg in untreated arteries) as well as the maximal VSM calcium concentration (60 mmHg vs. 120 mmHg in untreated vessels). These data suggest that ROS might be important in the normal maintenance of myogenic tone in control animals. In contrast, significant improvements in myogenic tone were observed in arteries from HSD rats following treatment with tiron and catalase where myogenic tone was significantly elevated compared to untreated arteries at 120 mmHg (Figure 4B). VSM calcium responses were significantly improved in arteries from both HSD and HFD following treatment with tiron and catalase (Figures 4B & C). The data in Figure 4B also show that tiron and catalase significantly increased the point at which the myogenic response reached a plateau in the HSD group (60 mmHg vs. 20 mmHg in untreated arteries) in addition to significantly increasing the maximum VSM calcium response (80 mmHg vs. 20 mmHg in untreated arteries). These findings demonstrate that ROS impair myogenic tone in HSD rats by depressing VSM calcium. Likewise, tiron and catalase significantly increased the maximum myogenic tone (60 mmHg vs. 40 mmHg in untreated arteries) and VSM calcium (100 mmHg vs. 80 mmHg) responses in arteries from HFD rats (Figure 4C). However, the myogenic tone was not significantly different from untreated HFD arteries at any point on the curve, showing that although VSM calcium levels were significantly increased, sensitivity to calcium remained attenuated in this group (Figure 4C).

**Figure 5:**
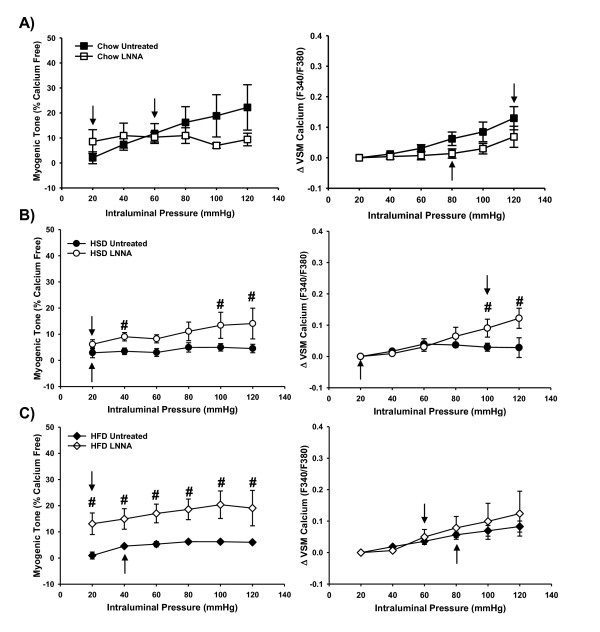
**Role of nitric oxide in the impaired myogenic response measured in arteries from Chowv (*n *= 6), HSD (*n *= 6) and HFD (*n *= 6) rats**. Experimental vessels were pre-treated with the general NOS inhibitor LNNA (100 μM). Data from untreated Chow, HSD and HFD arteries repeated from Figure 2 for comparison. Plateaus in responses at which point no further increases were seen are indicated by arrows and were determined from the two-way RM-ANOVA within group analyses. *#p *< 0.05 from untreated HSD or HFD control values at the same pressure step, respectively. Data expressed as mean ± SEM.

Inhibition of NOS using LNNA increased the overall tone of arteries from both HSD and HFD rats but did not increase the tone observed with incremental pressure steps (Figures 5B & C). A significant increase in VSM calcium levels with increasing pressure steps was seen in arteries from HSD rats pre-treated with LNNA but not in arteries from HFD or Chow rats (Figure 5). Pre-treatment of arteries from HSD rats did not affect the point where maximum myogenic tone was reached (20 mmHg) but did increase the maximum VSM calcium response (100 mmHg vs. 20 mmHg in untreated arteries; Figure 5B). In contrast, figure 5C shows a leftward shift of the maximum myogenic tone and VSM calcium responses in HFD arteries pre-treated with LNNA (Myogenic: 20 mmHg vs. 40 mmHg; Calcium: 60 mmHg vs. 80 mmHg in untreated arteries) suggesting a role for NO in the diminished myogenic responses observed in this group. Similar results were seen in the arteries from Chow rats (Figure 5A) wherein a leftward shift of the maximum VSM calcium responses (80 mmHg vs. 120 mmHg in untreated arteries) was accompanied by diminished myogenic tone (20 mmHg vs. 60 mmHg in untreated arteries). Since the endothelium was intact in these studies, it is possible that endothelial-derived factors or altered signaling may have contributed to the impaired myogenic tone in control arteries treated with LNNA. Combined inhibition of NOS and ROS did not further improve myogenic tone or VSM calcium responses in arteries from rats in any group (data not shown).

## Discussion

The major findings of this study are that 1) Myogenic tone is diminished in mesenteric arteries from rats fed either HSD or HFD (Figure 3) without qualitative changes in vascular morphology (Figures 1 and 2), 2) Decreased calcium sensitivity contributes to the impaired response in arteries from HFD rats (Figure 3), 3) Oxidative stress is involved in the impaired responses observed in arteries from HSD rats through decreasing VSM calcium levels (Figure 4B), 4) Nitric oxide and oxidative stress play roles in impaired myogenic tone in HFD arteries (Figures 4C &5C).

The results from the present study highlight changes that occur in vascular reactivity in overweight rats with impaired glucose tolerance [6]. Contrary to our hypothesis, this pre-diabetic condition did not result in enhanced myogenic tone in either special diet group (Figure 3). For HSD rats this corresponds with normal blood pressure (RESULTS) as expected in the absence of enhanced myogenic tone. In contrast, prior studies have shown that rats in the HFD group develop hypertension [7]. These findings differ from observations of obese and/or diabetic animals in which augmented myogenic tone contributes to their hypertensive state. For example, mesenteric arteries from the C57BL/KsJ-db/db mouse model of insulin-resistance have greater myogenic tone compared to wild-type mice [12]. Cerebral arteries from the BBZDR/Wor rat model of type 2 diabetes as well as the obese Zucker rat likewise show similar patterns of enhanced myogenic tone compared to lean control rats [13,14]. It is possible that the systemic hypertension observed in HFD rats is attributed to elevated circulating vasoconstrictors or augmented sympathetic outflow that are absent in the *ex vivo *setting or may act primarily in beds other than the mesenteric. It is also possible that the endothelium may have contributed to the impaired myogenic tone of the arteries in the current study and that removal of the endothelium may result in improved tone.

The roles of oxidative stress and NO in the response to increasing intraluminal pressure has been examined by others. Both O_2_^•- ^and H_2_O_2 _are associated with augmented myogenic tone in isolated mesenteric arteries from wild-type mice [15,16] and endothelial-derived NO opposes myogenic tone in mesenteric arteries [15,17]. Therefore, it would be expected that with increased oxidative stress contributing to diminished NO levels, myogenic tone would be greatly increased. However, despite contributing to impaired endothelin-1 mediated vasoconstriction [7], oxidative stress is also involved in the impaired myogenic tone observed in the HFD rats as evidenced by a rightward shift in the response following treatment of isolated arteries with tiron and catalase (Figure 4C). NO also contributes to the reduced myogenic response and VSM calcium levels in arteries from HFD rats (Figure 5C). Vasoconstrictor responses to endothelin-1 were not measured in the initial studies of HSD rats. Similar to the HFD rats, the current results show that NO does not play a role in the impaired myogenic tone seen in the vessels isolated from HSD rats (Figure 5B).

Myogenic tone has been shown to rely on L-type voltage operated calcium channels in small endothelium-intact mesenteric arteries (230-440 μm, inner diameter) isolated from male Wistar rats [18]. Similarly, myogenic responses in arteries from HSD rats are dependent on VSM calcium since restoration of levels with the antioxidants tiron and catalase were accompanied by improved myogenic tone (Figure 4B). In contrast, VSM calcium levels in isolated mesenteric arteries from HFD rats were comparable to the Chow-fed controls suggesting that sensitivity to calcium, as opposed to influx, is impaired in this group. Moreover, despite significantly increased VSM calcium following treatment of arteries with tiron and catalase, myogenic tone of arteries from HFD rats was not restored (Figure 4C).

The HSD and HFD rat models of increased adiposity respond differently to increases in intraluminal pressure from their obese or overtly diabetic counterparts. In this regard, the HSD and HFD animal models more closely mimic myogenic responses recorded for humans with type 2 diabetes. For example, small arteries (65-230 μm, inner diameter) dissected from gluteal fat biopsies taken from patients with type 2 diabetes or type 2 diabetes with hypertension showed significantly attenuated myogenic tone [19]. Goto-Kakizaki rat models of type 2 diabetes have been shown to develop increased tone in mesenteric arteries at 60 mmHg in comparison to normoglycemic Wistar rats. However, when these rats were fed a HFD (36% fat, 35% carbohydrate), the myogenic response was normalized to that of the control Wistar rats [20]. These findings are in agreement with those of the current study and point to the importance of diet in normal vascular function and suggest that the diet itself may regulate vascular reactivity.

## Conclusions

Rats fed either HSD or HFD develop impaired myogenic responses as a result of altered calcium signaling, although the mechanisms differ between the two models. For HSD rats, reduced tone is attributed to ROS-mediated reductions in VSM calcium levels. In contrast, ROS do not appear to be involved in the impaired myogenic tone observed in isolated mesenteric arteries from HFD rats. Rather, the impaired response in this group is likely due to reduced calcium sensitivity.

## Abbreviations

ANOVA: Analysis of variance; ACh: Acetylcholine; Chow: Normal rodent chow; H_2_O_2_: Hydrogen peroxide; HSD: High sucrose diet; HFD: High fat diet; LNNA: Nω-nitro-L-arginine; NO: Nitric oxide; NOS: Nitric oxide synthase; O_2_^•-^: Superoxide; PE: Phenylephrine; PSS: Physiological salt solution; ROS: Reactive oxygen species; Tiron: 4,5-dihydroxy-1,3-benzene-disulfonic acid; VSM: Vascular smooth muscle.

## Competing interests

The authors declare that they have no competing interests.

## Authors' contributions

KLS and BRW conceptualized and designed the study. KLS performed all experiments (with the exception of the morphological staining and imaging which was completed by Tamara Howard at The University of New Mexico), statistical analyses and wrote the first draft of the manuscript. BRW contributed to the writing of the manuscript, data presentation, interpretation and analyses. Both authors read and approved the final manuscript.
